# Supplementary material for: Meta-connectomic analysis maps consistent, reproducible, and transcriptionally relevant functional connectome hubs in the human brain
Source: Commun Biol. 2022 Oct 4;5:1056. doi: 10.1038/s42003-022-04028-x (PMC9532385; doi:10.1038/s42003-022-04028-x)
Supplement: Supplementary file 2 — Supplementary Information [file 42003_2022_4028_MOESM2_ESM.pdf]

Supplementary Information for

Meta-connectomic analysis maps consistent, reproducible, and transcriptionally-relevant functional connectome hubs in the human brain

Zhilei Xu, Mingrui Xia, Xindi Wang, Xuhong Liao, Tengda Zhao, Yong He

Corresponding author:

Yong He, Ph.D., E-mail: yong.he@bnu.edu.cn

Supplementary Information: 17 text pages, 11 figures.

Supplementary Note 1

Methodological variation or defects may cause controversial hub reports.

Methodological variation or defects in prior studies may cause controversial hub reports in specific regions, such as the primary areas, subcortical structures, and cerebellum. The controversy may arise from multifaceted sources.

Both unimodal and primary areas have been argued as candidate hub regions[1-6]. Our results demonstrated that functional connectome hubs were located in many unimodal cortices but not in the primary cortex. This finding is supported by the functional organization of the human cerebral cortex, where the primary cortex confines functional connectivity mostly in the primary cortex and portions of the unimodal cortex, whereas the unimodal cortex bridges the primary and heteromodal cortices[7, 8]. Prior study demonstrated that the primary cortex possesses most short-range functional connections[5], which could be systematically but spuriously elevated by subject head motion[9] or by unavoidable signal blurring across sulci or gyri[10]. These spurious short-range functional connections can be effectively suppressed by motion scrubbing, global signal regression, and stringent participant inclusion criteria as well as excluding short-range correlations[9-11], which all have been thoroughly addressed in the present study but were largely neglected in prior reports. Accordingly, it is reasonable to speculate that functional connectome hubs in the primary cortex reported in prior studies should be driven by spurious short-range functional connections.

Most subcortical structures have also been argued as candidate hubs, including the thalamus[2, 3, 12], basal ganglia[2, 13], amygdala[3, 13], and hippocampus[13]. Nevertheless, no subcortical structure was identified as a candidate hub in the present study. The inconsistency may be attributed to more complex sampling error and intercohort heterogeneity in subcortical structures. First, a prior report[14] demonstrated reliable estimation of subcortical-cortical functional connections requiring more data (~100 min per subject) than conventional quantities of rsfMRI data (5–20 min per subject) adopted by prior reports[2, 3, 12, 13]. In addition, individual features contribute to ~60% of the variance in subcortical-cortical functional connections[14], which is higher than ~35% in cortical-cortical[15] and ~45% in cerebellar-cortical[16] functional connections. It precludes reliable estimation of subcortical functional connections with only dozens of subjects. These two factors complicate both sampling error and intercohort heterogeneity in subcortical structures, which is in line with our observation of higher heterogeneity among cohorts in most subcortical structures than in the cortex (Supplementary Figure 1). In the random-effects meta-analysis framework, higher sampling error and intercohort heterogeneity will substantially undermine the effect size in subcortical structures, which may be the main reason of their absence as a candidate hub in the present study.

A portion of the cerebellum has also been argued as a candidate hub region[2, 3, 13]. Although the cerebellum was not included in the present study due to largely incomplete coverage during rsfMRI scanning in most cohorts, these reports conflicted with the metabolic pattern of the human brain. The cerebellum has been demonstrated to have the lowest level of aerobic glycolysis and a lower metabolic rate for glucose than most cortical regions[17], which makes it unfeasible for the cerebellum to maintain and run dense functional connections during rest. Combined with previous findings, we speculate that hub reports in the cerebellum should be

caused by inexplicable negative functional connections[13], spurious short-range functional connections[2, 3], or unstable estimation of functional connections due to inadequate data[16].

Functional connectome hubs have been reported in the superior temporal gyrus[1, 4-6, 13, 18], but rarely in the rolandic operculum. Our results demonstrated the superior temporal gyrus with subequal FCS levels compared with the rolandic operculum (Figure 2A). However, we identified significant hub peaks in the rolandic operculum and surrounding regions, such as the left area 43 and right 6r, rather than in the superior temporal gyrus. This finding may result from rsfMRI signal blurring across sulci because of the close proximity of the superior temporal gyrus and rolandic operculum[7]. Signal blurring may result in potential hub peaks in the superior temporal gyrus merging with those in the rolandic operculum and surrounding regions, causing false-negative observations of hub peaks in the superior temporal gyrus. Nevertheless, signal blurring is unavoidable in rsfMRI data processing, such as realignment, resampling, registration, and subject averaging[10]. Consequently, limited data resolution complicates the interpretation of functional connectome hubs in the superior temporal gyrus and rolandic operculum. This issue should be resolved in future studies using rsfMRI data with higher spatial resolution and greater signal specificity.

A prior study argued that identifying hub regions based on FCS highlights only members of large brain networks rather than brain regions playing crucial roles in global brain communication[10]. However, no significant correlation between a voxel's FCS and the size of the brain network to which it belongs could be identified in the present study (Supplementary Figure 4). The conclusion in the study[10] may be driven by unreasonable connection threshold (Pearson's r: 0.20-0.37) because we observed all hub peaks possessing substantial connections with Pearson's r less than 0.2 (Supplementary Figure 7).

We employed the FCS to identify hub regions because of its wide usage in prior studies[1-6, 12, 13, 18] and its high spatial similarity compared with other measures[19]. There are other useful hub identification measures, like the participation coefficient that may report more candidate hubs in subcortical structures because of their involvements in diverse functional domains, such as the basal ganglia[20] and the thalamus[21].

Supplementary Note 2

Global signal effect on spatial distribution of functional connectome hubs.

To examine the effect of global signal regression on hub distribution, we repeated identifying functional connectome hubs using preprocessed rsfMRI data without global signal regression. Supplementary Figure 10a shows that hub distribution was largely shifted by rsfMRI data preprocessing without global signal regression, of which some canonical hubs were absent, such as the inferior parietal gyrus, anterior insula, medial prefrontal cortex, and post cingulate cortex; more middle and anterior cingulate, visual, and auditory cortices were identified as hubs. This shifted hub distribution corresponds well with the report of brain regions with a global signal correlation significantly higher than the average[22]. It is compatible with our analysis of the global signal localization (GSL) score, which localizes the spatial distribution of the global signal (Supplementary Figure 10b). Specifically, for each individual, we computed the Fisher's z transformed Pearson's correlation coefficient between the global signal and preprocessed rsfMRI time series of each voxel. Next, we constructed a general linear model on these Fisher's z value maps within each cohort to reduce age and sex effects and performed a random-effects meta-analysis on these Fisher's z value maps across cohorts to address the across-cohort heterogeneity, resulting in a consistent GSL map (Supplementary Figure 10b). We observed that brain regions with GSL scores greater than 0.5 significantly overlapped with the shifted hub distribution (*Dice* = 0.833, $p < 0.001$, Supplementary Figure 10c, d).

Considering prior observations of a tight coupling between FCS and blood supply[23, 24], we examined differences between connectome hubs and non-hubs in metabolic measurements of blood supply (the cerebral blood flow) using the hub distribution in Supplementary Figure 10a. But one-sided Wilcoxon rank-sum test shown no significant difference between connectome hubs and non-hubs in the cerebral blood flow ($p = 0.077$, Supplementary Figure 10e).

Together, the hub distribution identified using preprocessed rsfMRI data without global signal regression was more likely derived from physiological artifacts rather than by the intrinsic or ongoing neuronal activity.

Supplementary References

1. Achard S, Salvador R, Whitcher B, Suckling J, Bullmore E. A Resilient, Low-Frequency, Small-World Human Brain Functional Network with Highly Connected Association Cortical Hubs. *The Journal of Neuroscience* **26**, 63-72 (2006).

2. Tomasi D, Volkow ND. Functional connectivity density mapping. *Proc Natl Acad Sci USA* **107**, 9885-9890 (2010).

3. Tomasi D, Volkow ND. Association between Functional Connectivity Hubs and Brain Networks. *Cerebral Cortex* **21**, 2003-2013 (2011).

4. de Pasquale F, *et al.* The connectivity of functional cores reveals different degrees of segregation and integration in the brain at rest. *NeuroImage* **69**, 51-61 (2013).

5. Liao X-H, *et al.* Functional brain hubs and their test–retest reliability: A multiband resting-state functional MRI study. *NeuroImage* **83**, 969-982 (2013).

6. Fransson P, Aden U, Blennow M, Lagercrantz H. The Functional Architecture of the Infant Brain as Revealed by Resting-State fMRI. *Cerebral Cortex* **21**, 145-154 (2011).

7. Yeo BT, *et al.* The organization of the human cerebral cortex estimated by intrinsic functional connectivity. *Journal of Neurophysiology* **106**, 1125-1165 (2011).

8. Sepulcre J, Sabuncu MR, Yeo TB, Liu H, Johnson KA. Stepwise Connectivity of the Modal Cortex Reveals the Multimodal Organization of the Human Brain. *The Journal of Neuroscience* **32**, 10649-10661 (2012).

9. Power JD, Barnes KA, Snyder AZ, Schlaggar BL, Petersen SE. Spurious but systematic correlations in functional connectivity MRI networks arise from subject motion. *NeuroImage* **59**, 2142-2154 (2012).

10. Power JD, Schlaggar BL, Lessov-Schlaggar CN, Petersen SE. Evidence for Hubs in Human Functional Brain Networks. *Neuron* **79**, 798-813 (2013).

11. Ciric R, *et al.* Benchmarking of participant-level confound regression strategies for the control of motion artifact in studies of functional connectivity. *NeuroImage* **154**, 174-187 (2017).

12. Dai Z, *et al.* Identifying and Mapping Connectivity Patterns of Brain Network Hubs in Alzheimer's Disease. *Cerebral Cortex* **25**, 3723-3742 (2014).

13. Cole MW, Pathak S, Schneider W. Identifying the brain's most globally connected regions. *NeuroImage* **49**, 3132-3148 (2010).

14. Greene DJ, *et al.* Integrative and Network-Specific Connectivity of the Basal Ganglia and Thalamus Defined in Individuals. *Neuron* **105**, 742-758 (2020).

15. Gratton C, *et al.* Functional Brain Networks Are Dominated by Stable Group and Individual Factors, Not Cognitive or Daily Variation. *Neuron* **98**, 439-452 (2018).

16. Marek S, *et al.* Spatial and Temporal Organization of the Individual Human Cerebellum. *Neuron* **100**, 977-993 (2018).

17. Vaishnavi SN, Vlassenko AG, Rundle MM, Snyder AZ, Mintun MA, Raichle ME. Regional aerobic glycolysis in the human brain. *Proc Natl Acad Sci USA* **107**, 17757-17762 (2010).

18. Buckner RL, *et al.* Cortical Hubs Revealed by Intrinsic Functional Connectivity: Mapping, Assessment of Stability, and Relation to Alzheimer's Disease. *The Journal of Neuroscience* **29**, 1860 (2009).

19. Wang X, Lin Q, Xia M, He Y. Differentially categorized structural brain hubs are involved in different microstructural, functional, and cognitive characteristics and contribute to individual identification. *Human Brain Mapping* **39**, 1647-1663 (2018).

20. Choi EY, Yeo BT, Buckner RL. The organization of the human striatum estimated by intrinsic functional connectivity. *Journal of Neurophysiology* **108**, 2242-2263 (2012).

21. Hwang K, Bertolero MA, Liu WB, D'Esposito M. The Human Thalamus Is an Integrative Hub for Functional Brain Networks. *The Journal of Neuroscience* **37**, 5594-5607 (2017).

22. Fox MD, Zhang D, Snyder AZ, Raichle ME. The Global Signal and Observed Anticorrelated Resting State Brain Networks. *Journal of Neurophysiology* **101**, 3270-3283 (2009).

23. Liang X, Zou Q, He Y, Yang Y. Coupling of functional connectivity and regional cerebral blood flow reveals a physiological basis for network hubs of the human brain. *Proc Natl Acad Sci USA* **110**, 1929-1934 (2013).

24. van den Heuvel MP, Sporns O. Network hubs in the human brain. *Trends in Cognitive Sciences* **17**, 683-696 (2013).

25. Kang HJ, *et al.* Spatio-temporal transcriptome of the human brain. *Nature* **478**, 483-489 (2011).

26. Harris Julia J, Jolivet R, Attwell D. Synaptic Energy Use and Supply. *Neuron* **75**, 762-777 (2012).

27. Burt JB, Helmer M, Shinn M, Anticevic A, Murray JD. Generative modeling of brain maps with spatial autocorrelation. *NeuroImage* **220**, 117038 (2020).

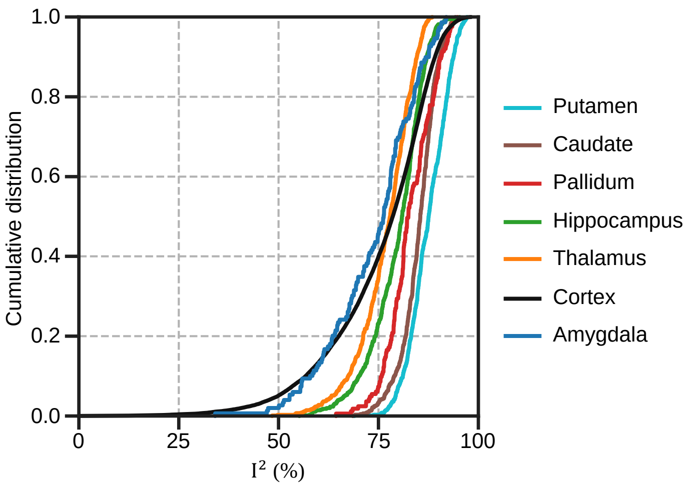

Supplementary Figure 1. Cumulative distribution function plot of I^2.

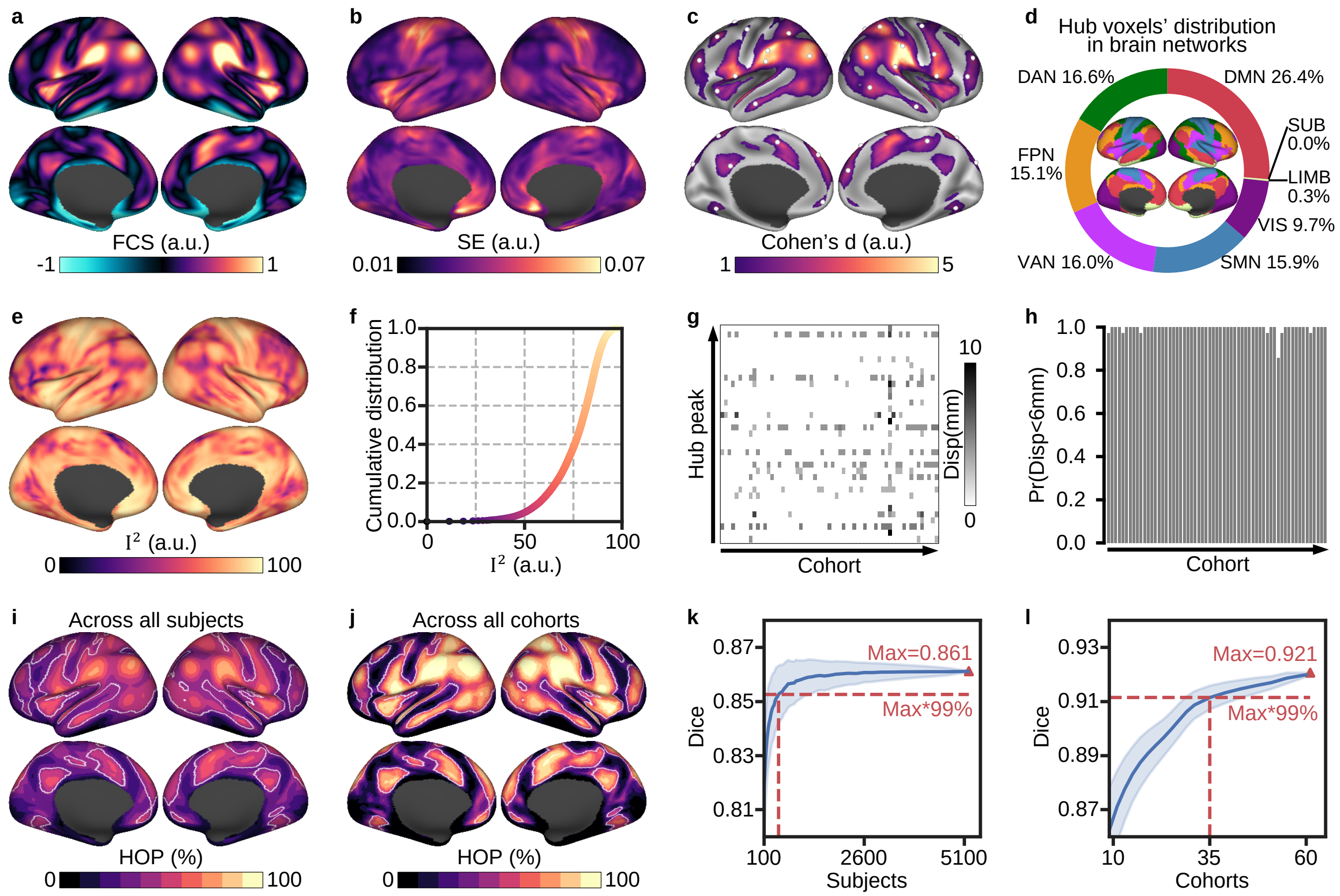

Supplementary Figure 2. Highly consistent and reproducible functional connectome hubs using a connection threshold of 0.05. a, **b** Robust FCS pattern (**a**) and its corresponding variance (standard error, SE) map (**b**) estimated using a harmonized voxelwise random-effects meta-analysis across 61 cohorts. **c** The most consistent functional connectome hubs (p < 0.001, cluster size > 200 mm^3). White spheres represent hub peaks. **d** Hub voxels' distribution in eight large-scale brain networks. Insets depicts the seven large-scale cortical networks[7]. **e** Heterogeneity measurement I^2 estimated through the random-effects meta-analysis. **f** Cumulative distribution function plot of I^2. **g** Heatmap of displacements of the 35 hub peaks after leaving one cohort out. **h** Bar plot of the probability across the 35 hub peaks whose displacement was less than 6 mm after leaving one cohort out. **i**, **j** Hub occurrence probability map (HOP) across all subjects (**i**) and all cohorts (**j**). White lines delineate boundaries of the identified hubs in **c**. **k**, **l** Dice's coefficient of the identified hubs in **c** compared with the top N (voxel number of the identified hubs in **c**) voxels with the highest hub occurrence probability values across randomly selected subjects (**k**) and randomly selected cohorts (**l**). Blue shading represents the standard deviation across 2,000 random selections. a.u., arbitrary unit.

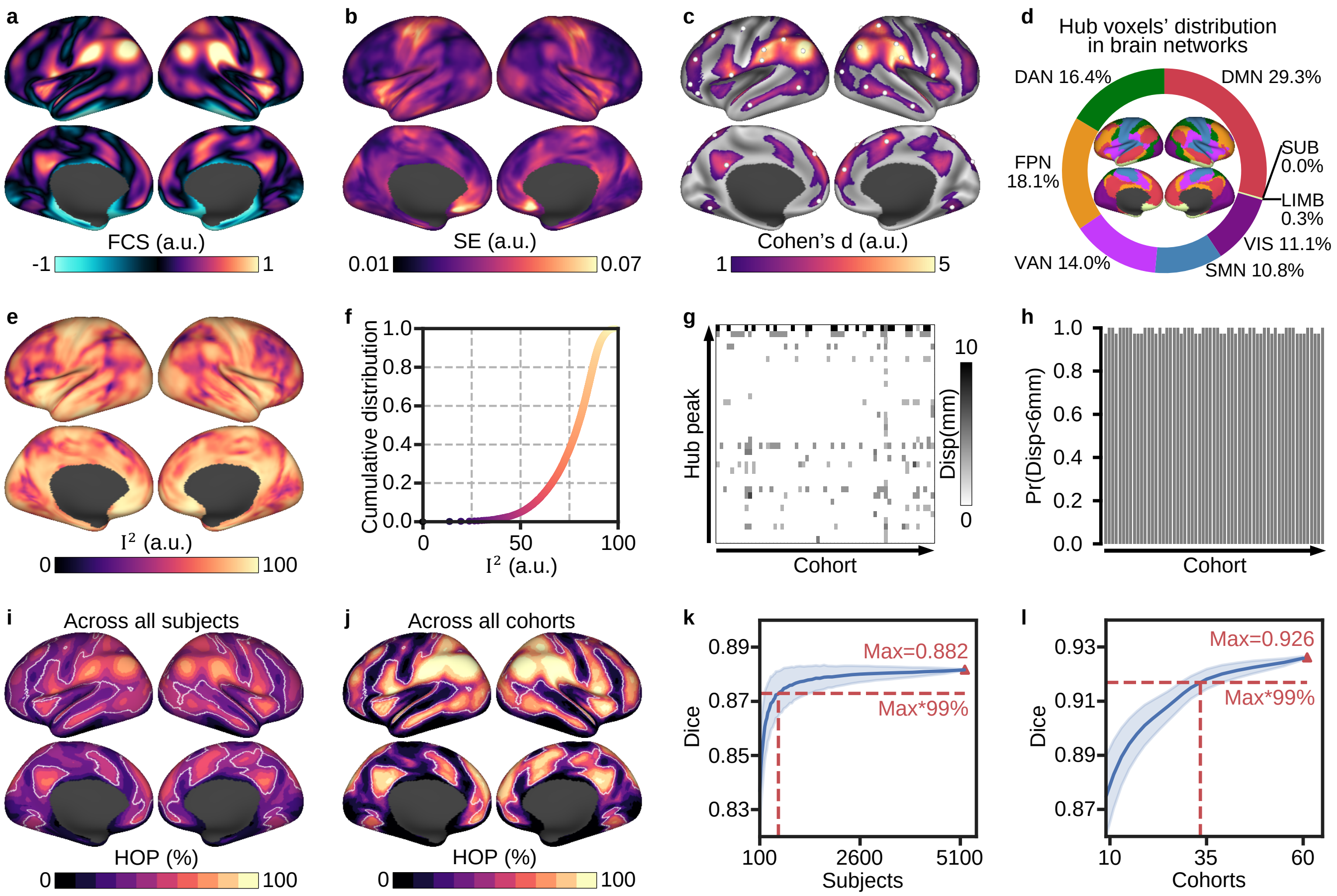

Supplementary Figure 3. Highly consistent and reproducible functional connectome hubs using a connection threshold of 0.2. a, **b** Robust FCS pattern (**a**) and its corresponding variance (standard error, SE) map (**b**) estimated using a harmonized voxelwise random-effects meta-analysis across 61 cohorts. **c** The most consistent functional connectome hubs (p < 0.001, cluster size > 200 mm^3). White spheres represent hub peaks. **d** Hub voxels' distribution in eight large-scale brain networks. Insets depicts the seven large-scale cortical networks[7]. **e** Heterogeneity measurement I^2 estimated through the random-effects meta-analysis. **f** Cumulative distribution function plot of I^2. **g** Heatmap of displacements of the 35 hub peaks after leaving one cohort out. **h** Bar plot of the probability across the 35 hub peaks whose displacement was less than 6 mm after leaving one cohort out. **i**, **j** Hub occurrence probability map (HOP) across all subjects (**i**) and all cohorts (**j**). White lines delineate boundaries of the identified hubs in **c**. **k**, **l** Dice's coefficient of the identified hubs in **c** compared with the top N (voxel number of the identified hubs in **c**) voxels with the highest hub occurrence probability values across randomly selected subjects (**k**) and randomly selected cohorts (**l**). Blue shading represents the standard deviation across 2,000 random selections. a.u., arbitrary unit.

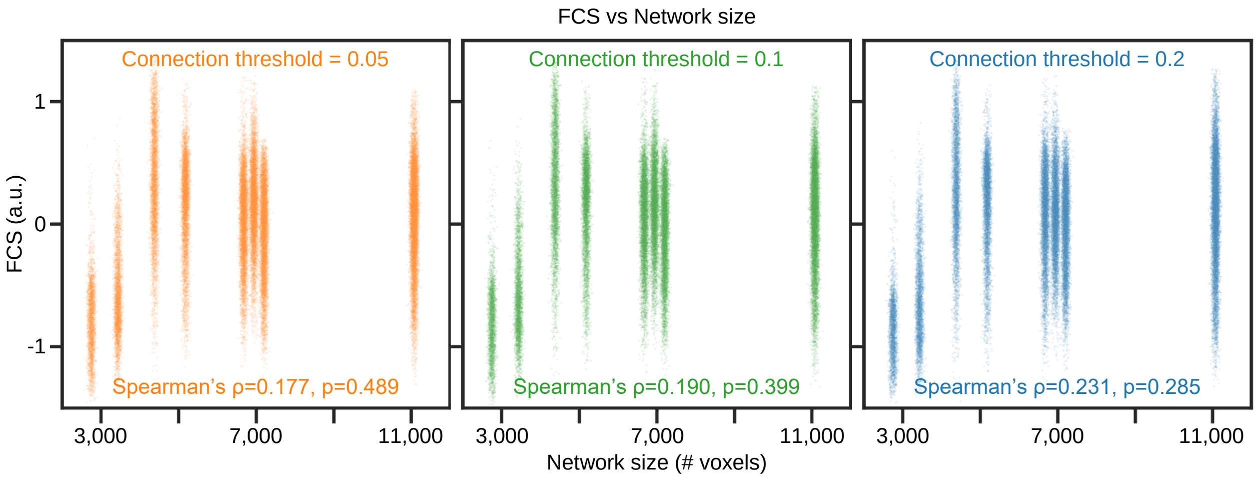

Supplementary Figure 4. Relationship between FCS and network size. Scatter plot showing no significant correlation between the FCS of voxels and the size of the brain network to which they belong. The *p* value was estimated through a nonparametric permutation test with 10,000 iterations and was Bonferroni-corrected. For each iteration, the voxel number of each network was reshuffled. Each dot represents a voxel. For illustration purposes, dots were jittered along the x axis (uniform jitter of ±50 voxels). a.u., arbitrary unit.

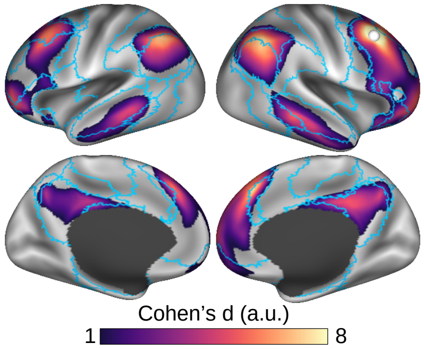

Supplementary Figure 5. Functional connectivity profile of the right 8Av region. White spheres represent the right 8Av seed (MNI coordinates: 45, 18, 45). Blue lines delineate boundaries of the seven cortical networks shown in Figure 1d. a.u., arbitrary unit.

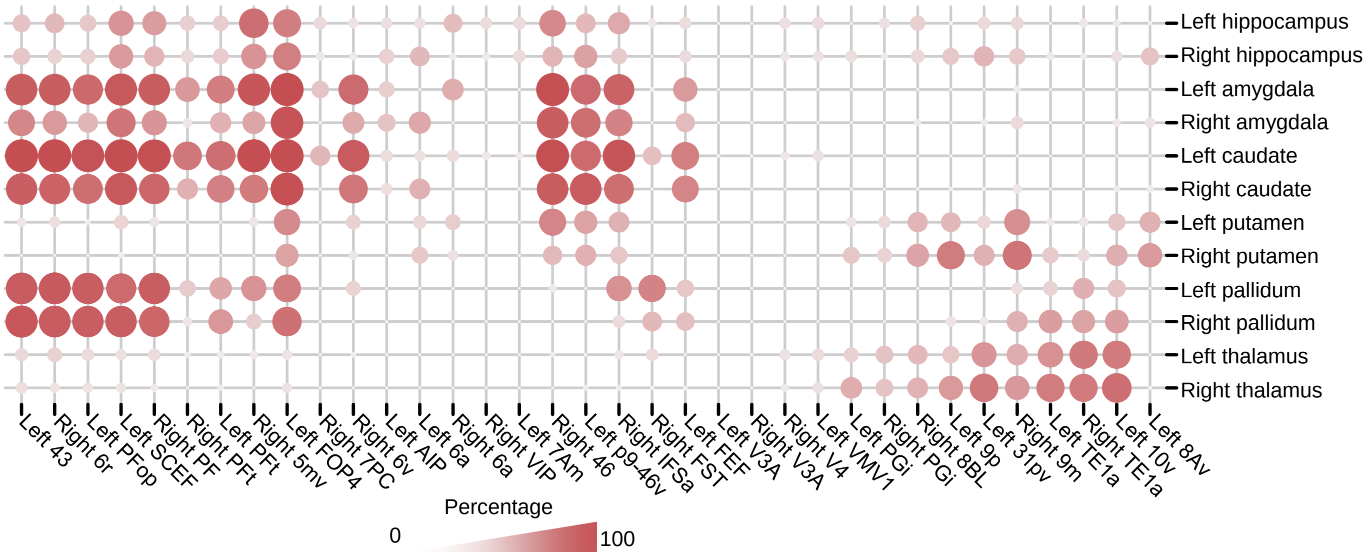

Supplementary Figure 6. Brain hubs' connectivity profiles with subcortical nucleus. Percentage matrix showing brain hubs' heterogeneous connectivity profiles with subcortical nucleus. Each item of the percentage matrix represents the voxel percentage of one subcortical nucleus connected with one hub.

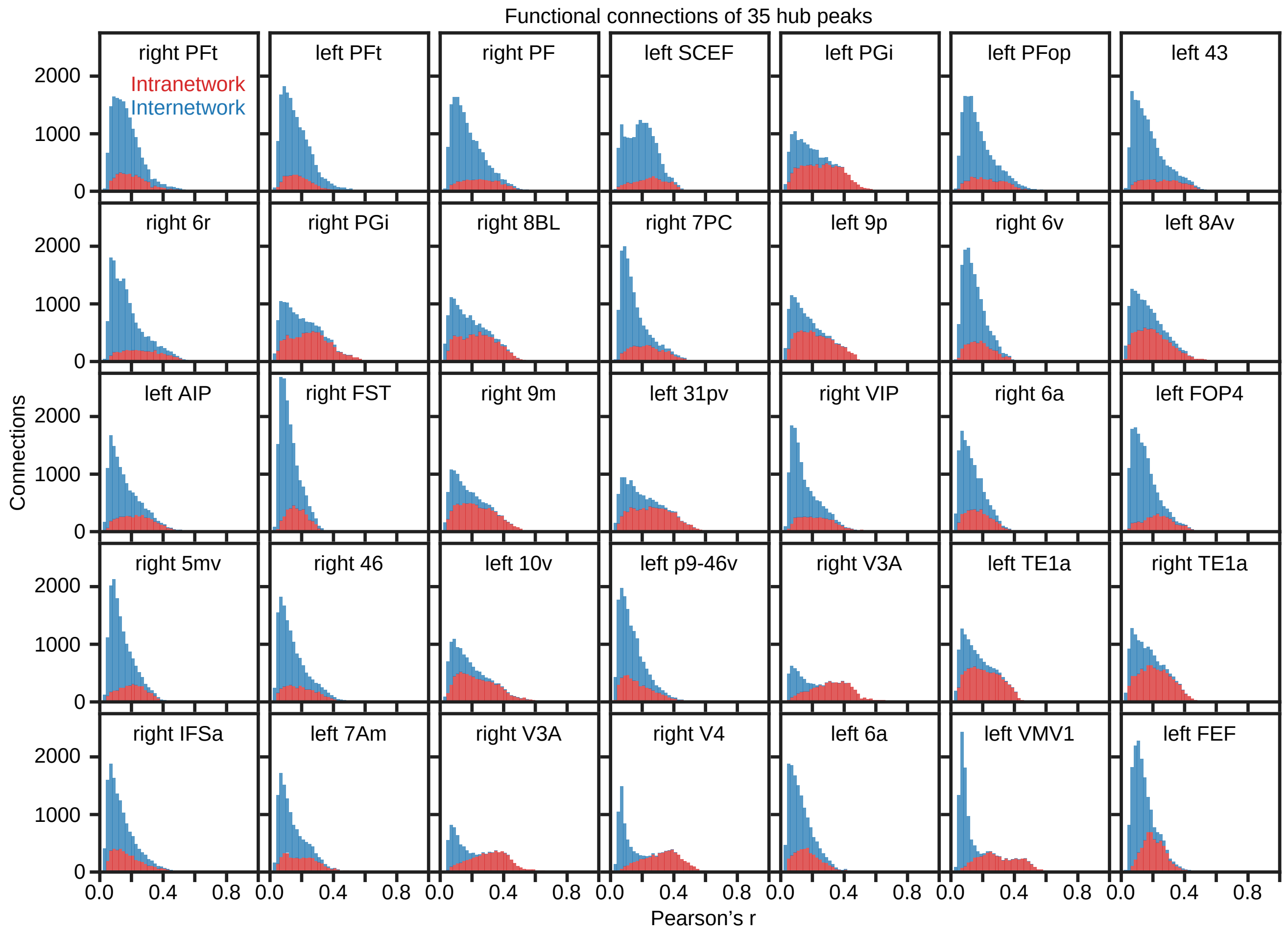

Supplementary Figure 7. Histogram plot of the connection strength of each hub's robust functional connectivity map shown in Figure 3. Intranetwork and iternetwork connections are displayed as stacked.

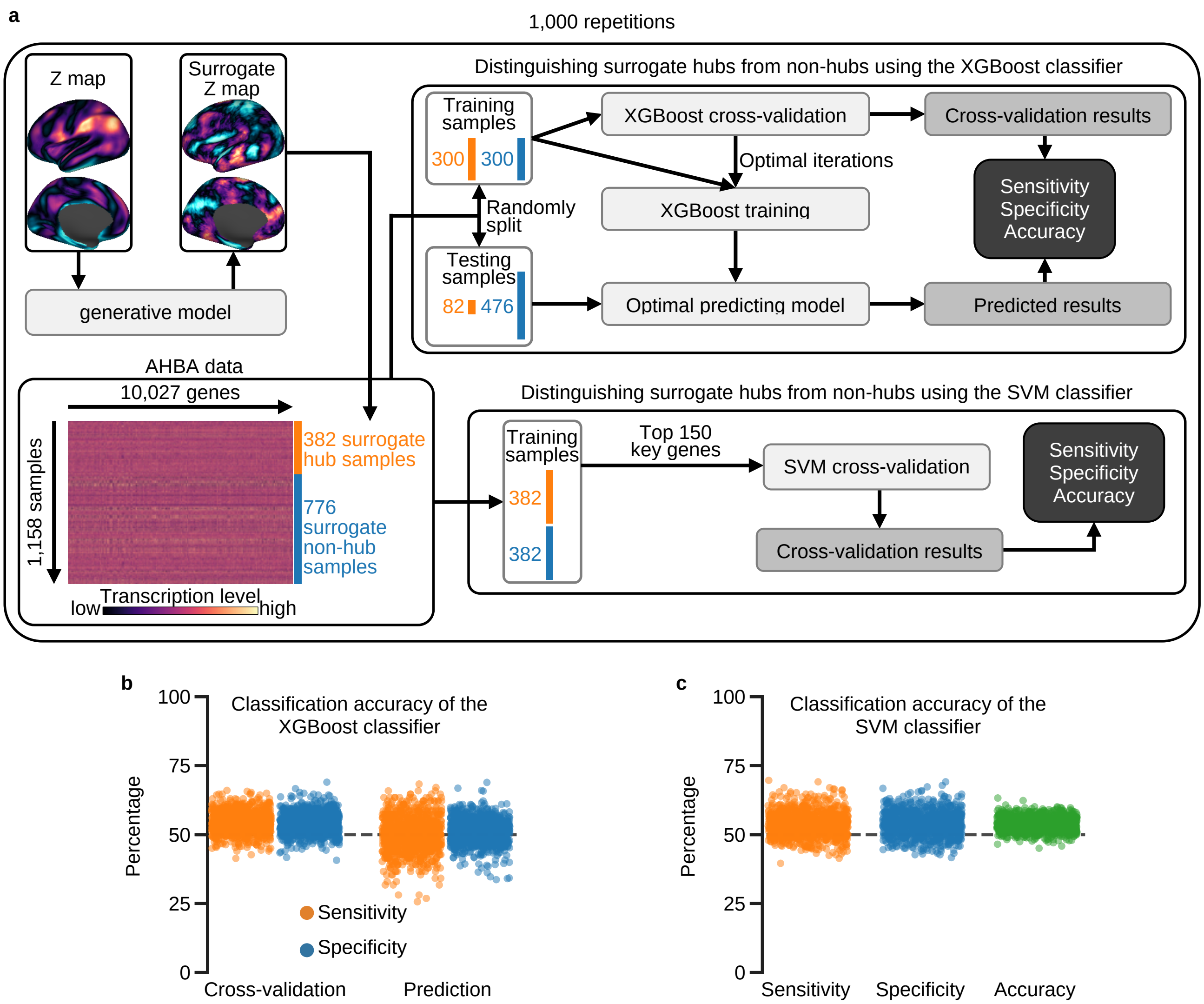

Supplementary Figure 8. Transcriptomic data cannot distinguish surrogate hubs from surrogate non-hubs. a Schematic diagram of generating surrogate hub identification and using XGBoost and SVM classifiers to distinguish surrogate hub samples from surrogate non-hub samples. **b**, **c** Performance of the XGBoost and SVM classifier. Each dot represents one repetition in **a**. The horizontal gray dashed line represents the chance level accuracy rate (50%).

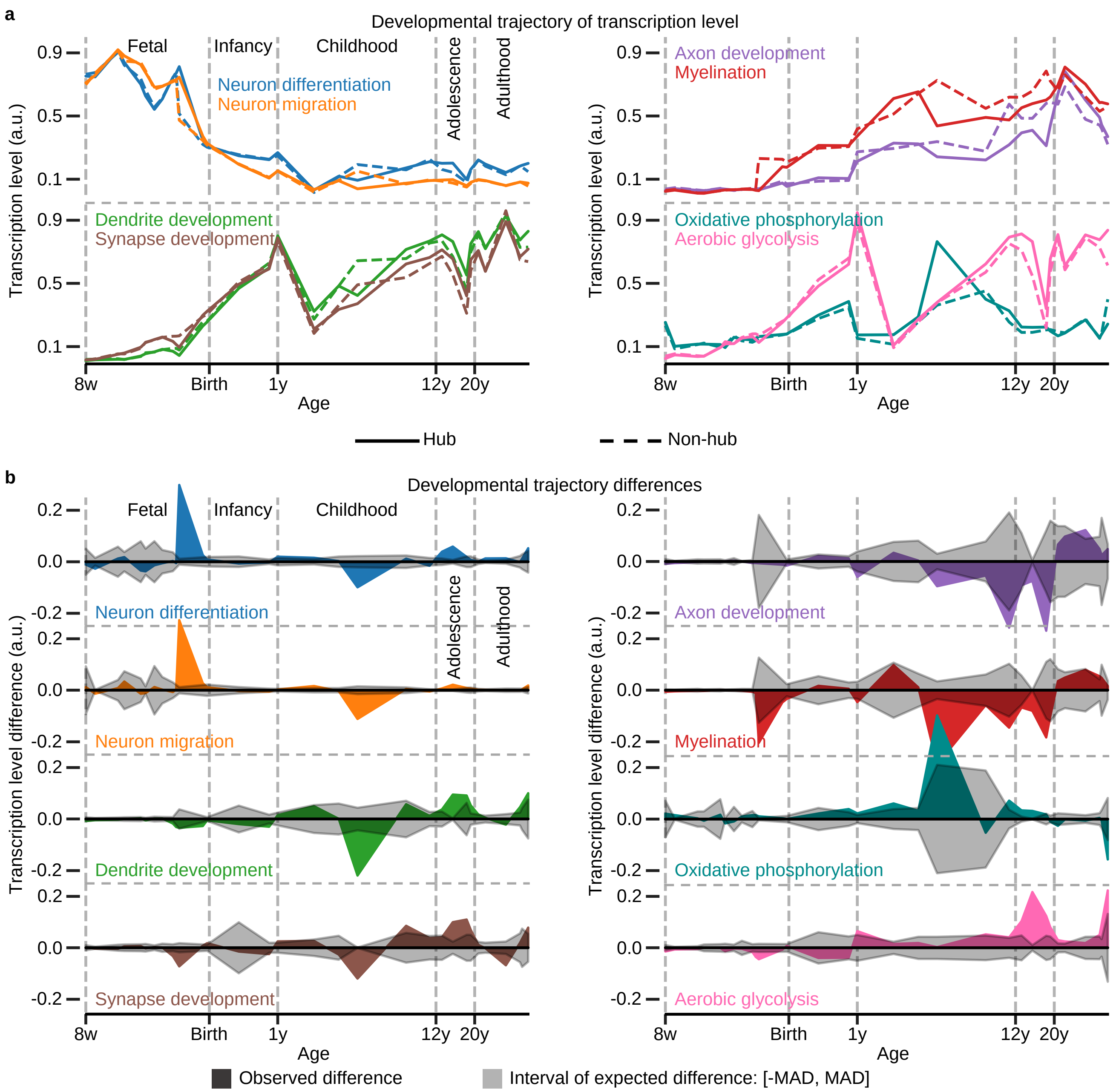

Supplementary Figure 9. Analysis of developmental trajectory of transcription level using only neocortical regions. a Developmental trajectory of transcription level in hub and non-hub regions for genes involved in key neurodevelopmental processe[25] and main neuronal metabolic pathways[26]. **b** Differences in the developmental trajectory of transcription level between hub and non-hub regions shown in **a**. MAD, the median absolute deviation of transcription level across brain regions. w, post-conceptional week; y, postnatal year; a.u., arbitrary unit.

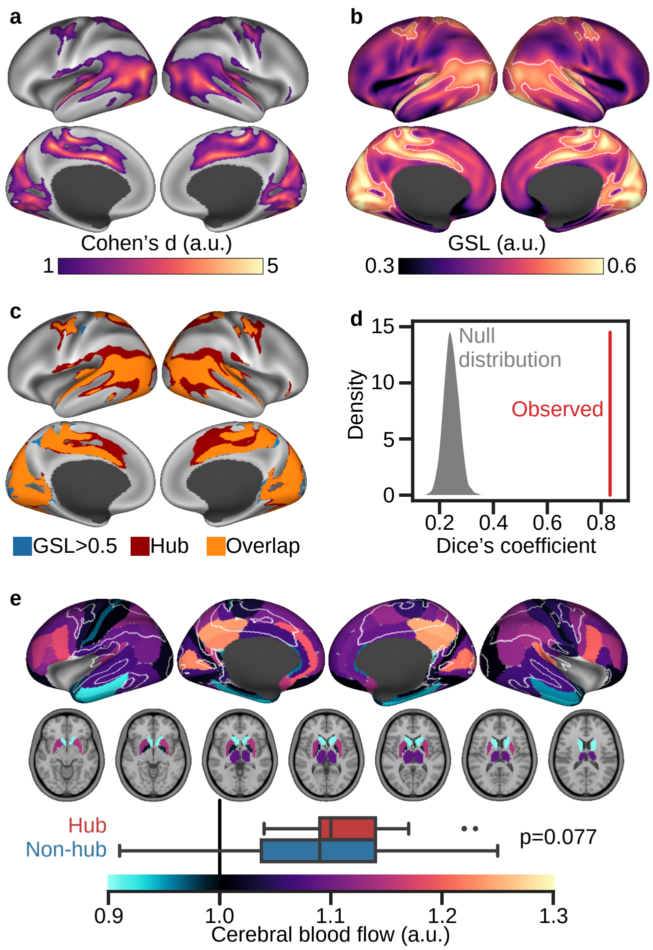

Supplementary Figure 10. Global signal effect on spatial distribution of functional connectome hubs. a Functional connectome hubs identified using preprocessed rsfMRI data without global signal regression. Brain regions with FCS significantly higher than zero were identified as hubs ($p < 0.001$, cluster size > 200 mm^3). **b** Global signal localization (GSL) score distribution. Brain regions with GSL scores greater than 0.5 were delineated with white lines. **c** Overlap of brain regions with GSL scores greater than 0.5 on the identified hubs in **a**. **d** Dice's coefficient obtained by comparing brain regions with GSL scores greater than 0.5 with the identified hubs in **a** and its corresponding null distribution that was constructed by generating 1,000 surrogate maps of the hub distribution map in **a** with the spatial autocorrelations being corrected using a generative model[27]. **e** Difference in cerebral blood flow between the identified hub and non-hub regions in **a**. The cerebral blood flow of 82 Brodmann areas and seven subcortical structures were provided by a prior study[17]. White lines delineate boundaries of the identified hubs in **a**. Boxplot left and right edges, vertical black lines, and whiskers and dots depict the 25th and 75th percentiles, median, and extreme nonoutlier and outlier values, respectively. Brodmann areas with more than 50% vertices or subcortical structures with more than 50% voxels identified as hubs were regarded as hub regions (n=21), vice versa as non-hub regions (n=68). The statistical significance of one-sided Wilcoxon rank-sum test was determined by 1,000 permutation tests. a.u., arbitrary unit.

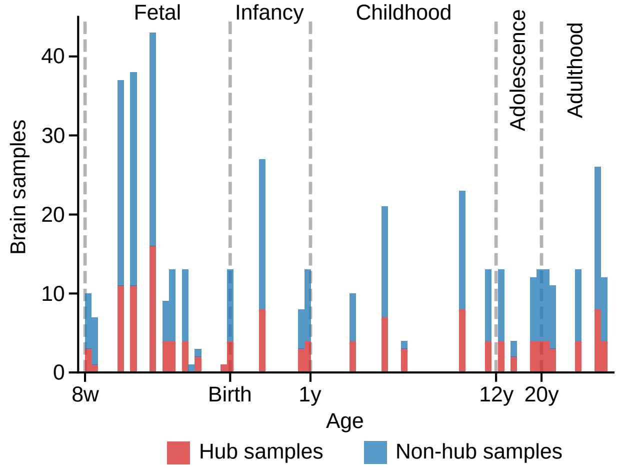

Supplementary Figure 11. Age distribution of brain samples from the BrainSpan Atlas dataset. Hub and non-hub samples are displayed as stacked. w, post-conceptional week; y, postnatal year.
