# Supplementary material for: Meta-connectomic analysis maps consistent, reproducible, and transcriptionally relevant functional connectome hubs in the human brain
Source: Commun Biol. 2022 Oct 4;5:1056. doi: 10.1038/s42003-022-04028-x (PMC9532385; doi:10.1038/s42003-022-04028-x)
Supplement: Supplementary file 3 — Description of Additional Supplementary Files [file 42003_2022_4028_MOESM3_ESM.pdf]

Description of Additional Supplementary Files

File name: Supplementary Data 1

Description: Genes' contributions to the XGBoost classifier.

File name: Supplementary Data 2

Description: GOrilla GO enrichment analysis results for the top 150 key genes.

File name: Supplementary Data 3

Description: GOrilla GO enrichment analysis results for the ranked 10,027 genes.

File name: Supplementary Data 4

Description: DAVID GO enrichment analysis results for the top 150 key genes.

File name: Supplementary Data 5

Description: DAVID disease association analysis results for the top 150 key genes.

File name: Supplementary Data 6

Description: Genes associated with key neurodevelopment processes.

File name: Supplementary Data 7

Description: Genes associated with main neuronal metabolic pathways.

File name: Supplementary Data 8

Description: Included cohorts in the final analysis.

File name: Supplementary Data 9

Description: Included AHBA samples in the final analysis.

File name: Supplementary Data 10

Description: Included BrainSpan samples in the final analysis
