# Supplementary material for: Meta-connectomic analysis maps consistent, reproducible, and transcriptionally relevant functional connectome hubs in the human brain
Source: Commun Biol. 2022 Oct 4;5:1056. doi: 10.1038/s42003-022-04028-x (PMC9532385; doi:10.1038/s42003-022-04028-x)
Supplement: Supplementary file 5 — Reporting Summary [file 42003_2022_4028_MOESM5_ESM.pdf]

nature portfolio

Corresponding author(s): Yong He

Last updated by author(s): Sep 23, 2022

Reporting Summary

Nature Portfolio wishes to improve the reproducibility of the work that we publish. This form provides structure for consistency and transparency in reporting. For further information on Nature Portfolio policies, see our Editorial Policies and the Editorial Policy Checklist.

Statistics

For all statistical analyses, confirm that the following items are present in the figure legend, table legend, main text, or Methods section.

n/a	Confirmed	
☐	☒	The exact sample size (*n*) for each experimental group/condition, given as a discrete number and unit of measurement
☐	☒	A statement on whether measurements were taken from distinct samples or whether the same sample was measured repeatedly
☐	☒	The statistical test(s) used AND whether they are one- or two-sided<br>*Only common tests should be described solely by name; describe more complex techniques in the Methods section.*
☐	☒	A description of all covariates tested
☐	☒	A description of any assumptions or corrections, such as tests of normality and adjustment for multiple comparisons
☐	☒	A full description of the statistical parameters including central tendency (e.g. means) or other basic estimates (e.g. regression coefficient) AND variation (e.g. standard deviation) or associated estimates of uncertainty (e.g. confidence intervals)
☐	☒	For null hypothesis testing, the test statistic (e.g. *F*, *t*, *r*) with confidence intervals, effect sizes, degrees of freedom and *P* value noted<br>*Give P values as exact values whenever suitable.*
☒	☐	For Bayesian analysis, information on the choice of priors and Markov chain Monte Carlo settings
☒	☐	For hierarchical and complex designs, identification of the appropriate level for tests and full reporting of outcomes
☐	☒	Estimates of effect sizes (e.g. Cohen's *d*, Pearson's *r*), indicating how they were calculated

Our web collection on statistics for biologists contains articles on many of the points above.

Software and code

Policy information about availability of computer code

Data collection	No software was used in data collection.
Data analysis	The code to reproduce the results and visualizations of this manuscript is available at https://doi.org/10.5281/zenodo.7106831. Software packages used in this manuscript include MATLAB R2013a (https://www.mathworks.com/products/matlab.html), SPM12 v6470 (https://www.fil.ion.ucl.ac.uk/spm/software/spm12), GRETNA v2.0.0 (https://www.nitrc.org/projects/gretna), Connectome Workbench v1.4.2 (https://www.humanconnectome.org/software/connectome-workbench), cifti-matlab v2 (https://github.com/Washington-University/cifti-matlab), R 4.0.2 (https://www.r-project.org), XGBoost package v1.2.0.1 (https://cran.r-project.org/web/packages/xgboost), Python 3.8.3 (https://www.python.org), and scikit-learn package v0.23.2 (https://scikit-learn.org). Online analysis tools used in this manuscript include GOrilla (http://cbl-gorilla.cs.technion.ac.il) and DAVID v6.8 (https://david.ncifcrf.gov).

For manuscripts utilizing custom algorithms or software that are central to the research but not yet described in published literature, software must be made available to editors and reviewers. We strongly encourage code deposition in a community repository (e.g. GitHub). See the Nature Portfolio guidelines for submitting code & software for further information.

Data

Policy information about availability of data

All manuscripts must include a data availability statement. This statement should provide the following information, where applicable:

- Accession codes, unique identifiers, or web links for publicly available datasets
- A description of any restrictions on data availability
- For clinical datasets or third party data, please ensure that the statement adheres to our policy

The MRI data of the first 60 cohorts listed in Supplementary Data 8 are available at the International Neuroimaging Data-sharing Initiative (http://fcon_1000.projects.nitrc.org), Brain Genomics Superstruct Project (https://doi.org/10.7910/DVN/25833), Human Connectome Project (https://www.humanconnectome.org), MPI-Leipzig Mind-Brain-Body Project (https://openneuro.org/datasets/ds000221), and Age-ility Project (https://www.nitrc.org/projects/age-ility). The MRI data of the PKU cohort are under active use by the reporting laboratory and will be available from the corresponding author upon reasonable request. The preprocessed AHBA dataset is available at https://doi.org/10.6084/m9.figshare.6852911. The normalized BrainSpan Atlas dataset is available at http://brainspan.org/static/download.html. The neurotransmitter receptor and transporter density maps provided by the JuSpace toolbox are available at https://github.com/juryxy/JuSpace. The fiber length profiling dataset is available at https://balsa.wustl.edu/study/1K3l. The cortical thickness atrophy dataset provided by the ENIGMA Toolbox is available at https://github.com/MICA-MNI/ENIGMA. Numerical source data to reproduce all figure panels is available at https://doi.org/10.6084/m9.figshare.21194128.

Human research participants

Policy information about studies involving human research participants and Sex and Gender in Research.

Reporting on sex and gender	Male 2377, female 2835.<br>No new data was collected for this manuscript. Sex information was provided by the existing datasets.
Population characteristics	Age and sex data provided by existing dataset were used in this manuscript. Age: 18-36 years. 2377 males, 2835 femals.
Recruitment	Recruitment is not appropriate to this study, because no new data was collected for this manuscript.
Ethics oversight	No new data was collected for this manuscript. We collected rsfMRI dataset from public data-sharing platforms and existing in-house cohorts, which consists of 73 cohorts from Asia, Europe, North America, and Australia. Data of each cohort were collected with participants' written informed consent and with approval by the respective local institutional review boards.

Note that full information on the approval of the study protocol must also be provided in the manuscript.

Field-specific reporting

Please select the one below that is the best fit for your research. If you are not sure, read the appropriate sections before making your selection.

☒ Life sciences ☐ Behavioural & social sciences ☐ Ecological, evolutionary & environmental sciences

For a reference copy of the document with all sections, see nature.com/documents/nr-reporting-summary-flat.pdf

Life sciences study design

All studies must disclose on these points even when the disclosure is negative.

Sample size	No a-prior sample size was calculated, but we included a large-sample resting-state fMRI dataset of 5,212 healthy young adults (aged 18–36 years) in this manuscript. Our validation analysis in this manuscript demonstrated that resting-state fMRI dataset of 5,212 subjects were adequate to obtain highly reproducible results.
Data exclusions	For MRI data of the all 73 cohorts, we used strict and uniform inclusion criteria with regard to structural abnormality and head motion. Specifically, we first reviewed T1-weighted structural MRI data for all participants and excluded subjects with identifiable lesion or structural abnormality (e.g., regional atrophy and posterior cranial fossa arachnoid cyst). Then, during resting-statefMRI data preprocessing, we excluded subjects with significant head motion (translation above 3 mm or rotation above 3° in any direction) and subjects with more than 25% interpolated volumes after head motion scrubbing. To reduce the potential effects of development and aging on our results, we restricted our analysis to healthy young adults (aged 18–36 years). To ensure sufficient statistical power, twelve cohorts were discarded due to having fewer than 10 participants that passed quality controls. After these stringent quality controls, we included preprocessed resting-state fMRI data of 5,212 healthy young adults (2,377 males) from 61 cohorts in the final analysis.
Replication	Reproducibility of main findings was ensured by extensive validation analysis of analysis parameters, analysis models and tools, and datasets.
Randomization	Randomization is not appropriate to this study, because no new data was collected for this manuscript.
Blinding	Blinding is not appropriate to this study, because no new data was collected for this manuscript.

Reporting for specific materials, systems and methods

We require information from authors about some types of materials, experimental systems and methods used in many studies. Here, indicate whether each material, system or method listed is relevant to your study. If you are not sure if a list item applies to your research, read the appropriate section before selecting a response.

Materials & experimental systems

n/a	Involved in the study
☒	☐ Antibodies
☒	☐ Eukaryotic cell lines
☒	☐ Palaeontology and archaeology
☒	☐ Animals and other organisms
☒	☐ Clinical data
☒	☐ Dual use research of concern

Methods

n/a	Involved in the study
☒	☐ ChIP-seq
☒	☐ Flow cytometry
☐	☒ MRI-based neuroimaging

Magnetic resonance imaging

Experimental design

Design type	Resting-state.
Design specifications	Resting-state.
Behavioral performance measures	None.

Acquisition

Imaging type(s)	Resting-state functional MRI, T1-weighted structural MRI.
Field strength	Varying, listed in Supplementary Data 8.
Sequence & imaging parameters	Varying, listed in Supplementary Data 8.
Area of acquisition	Whole brain.

Diffusion MRI ☐ Used ☒ Not used

Preprocessing

Preprocessing software	Functional MRI data were preprocessed routinely using SPM12 v6470 (https://www.fil.ion.ucl.ac.uk/spm/software/spm12) and GRETNA v2.0.0 (https://www.nitrc.org/projects/gretna). Functional MRI data were spatially smoothed using a 6-mm full-width at half-maximum Gaussian kernel.
Normalization	Functional MRI data were non-linear normalized to the MNI space using SPM12.
Normalization template	EPI template in the MNI space provided by SPM12.
Noise and artifact removal	Functional MRI data: Nuisance variables of linear trend, 24 head motion parameters, average white matter, cerebrospinal fluid, and global brain signals were removed using regression. Band-pass filtering between 0.01 to 0.1 Hz was applied.
Volume censoring	Volumes with framewise displacement exceeding 0.5 mm and their adjacent volumes (1 back and 2 forward) were replaced with linearly interpolated data using GRETNA.

Statistical modeling & inference

Model type and settings	We used a general linear model within each cohort to reduce age and gender effects. We used a random-effects meta-analysis model to address the across-cohort heterogeneity.
Effect(s) tested	Effect size was estimated using Cohen's d metric. No ANOVA or factorial design was used.

Specify type of analysis: ☒ Whole brain ☐ ROI-based ☐ Both

Statistic type for inference (See Eklund et al. 2016)	Voxel-wise.

Correction	Permutation and Bonferroni correction.

Models & analysis

n/a	Involved in the study
☐	☒ Functional and/or effective connectivity
☐	☒ Graph analysis
☐	☒ Multivariate modeling or predictive analysis

Functional and/or effective connectivity	Pearson's correlation.
Graph analysis	Functional connectivity strength.
Multivariate modeling and predictive analysis	We trained supervised machine learning classifiers based on XGBoost and SVM to distinguish connectome hubs from non-hubs using 10,027 genes' transcriptomic features from the preprocessed AHBA dataset. The sensitivity, specificity, and accuracy rate of the classifier were stably estimated by repeating the training and testing procedure 1,000 times. Cross-validation was used to obtain the optimal prediction model.
